# Inhibition of α-, β-, γ-, δ-, ζ- and η-class carbonic anhydrases from bacteria, fungi, algae, diatoms and protozoans with famotidine

**DOI:** 10.1080/14756366.2019.1571273

**Published:** 2019-02-07

**Authors:** Andrea Angeli, Mariana Pinteala, Stelian S. Maier, Sonia Del Prete, Clemente Capasso, Bogdan C. Simionescu, Claudiu T. Supuran

**Affiliations:** a Dipartimento Neurofarba, Sezione di Scienze Farmaceutiche e Nutraceutiche, Università degli Studi di Firenze, Florence, Italy;; b Centre of Advanced Research in Bionanoconjugates and Biopolymers Department, “Petru Poni” Institute of Macromolecular Chemistry, Iasi, Romania;; c “Gheorghe Asachi” Technical University of Iasi, Polymers Research Center, Polymeric Release Systems Research Group, Iasi, Romania;; d Istituto di Bioscienze e Biorisorse, CNR, Napoli, Italy

**Keywords:** Carbonic anhydrase, bacterial/fungal/diatom/protozoan enzymes, *Helicobacter pylori*, *Vibrio cholerae*, *Burkholderia pseudomallei*, *Plasmodium falciparum*

## Abstract

Famotidine, an antiulcer drug belonging to the H_2_ antagonists class of pharmacological agents, was recently shown to potently inhibit human (h) and bacterial carbonic anhydrases (CAs, EC 4.2.1.1). We investigated the inhibitory effects of famotidine against all classes of CAs from the pathogenic bacteria *Vibrio cholerae*, *Burkholderia pseudomallei* and *Mycobacterium tuberculosis* Rv3273 β-CA, as well as the CAs from the nonpathogenic bacteria/cyanobacteria *Sulfurihydrogenibium yellowstonensis*, *S. azorense*, *Pseudoalteromonas haloplanktis*, *Colwellia psychrerythraea* and *Nostoc commune*. The δ- and ζ-CAs from the diatom *Thalassiosira weissflogii*, the fungal enzymes from *Cryptococcus neoformans*, *Candida glabrata* and *Malassezia globosa*, as well as the protozoan enzymes from *Trypanosoma cruzi* and *Plasmodium falciparum*, were also investigated. *Anopheles gambiae* β-CA was also investigated. All these enzymes were effectively inhibited by famotidine, with affinities between the low nanomolar to the micromolar range. The best inhibition was observed against *C. glabrata* β-CA and TweCAζ, with K_I_s ranging between 13.6 and 22.1 nM.

## 1. Introduction

Carbonic anhydrases (CAs, EC 4.2.1.1) are ubiquitous metalloenzymes all over the phylogenetic tree, with seven distinct genetic families described so far, the α-, β-, γ-, δ-, ζ-, η- and Θ-CAs^1–13^, and the probability to discover other such families is quite high. This is mainly due to the fact that these enzymes catalyse a simple but essential chemical reaction, the interconversion of carbon dioxide and water, with formation of bicarbonate and protons: CO_2_+H_2_O ⇌ HCO_3_^–^+H^+^^1^^,^^14–18^. In tissues/organisms where CAs are present, they are involved in pH regulation (a tightly controlled process in all living organisms) and metabolic processes connected to carboxylation/decarboxylation reactions^1–13^. In plants and some bacteria (e.g. cyanobacteria), CAs also play a function in photosynthesis, for example assuring a carbon concentrating mechanism (CCM) which enhances the concentration of CO_2_ available for 1,5-bisphosphate carboxylase/oxygenase (RUBISCO)^19–22^. Diatoms are also encoding for several classes of CAs. In fact, the δ- and ζ-CAs were discovered in these organisms^4^^,^^6^, in which they probably play essential functions, poorly understood at this moment^21^.

In the genome of many pathogenic bacteria (e.g. *Helicobacter pylori*, the Gram-negative bacterium responsible of gastric ulcers^10^; *Vibrio cholerae*, the Gram-negative bacterium provoking cholera)^9^, *Brucella suis*, the non-motile Gram-negative coccobacillus responsible of brucellosis^1^^,^^2^, *Mycobacterium tuberculosis*, the obligate pathogenic bacterium responsible for tuberculosis^8^, *Burkholderia pseudomalei*^14^, and many others) CAs belonging to at least three classes were found, and many of them were shown to be essential for growth of the pathogen^17–19^. CA inhibitors (CAIs) targeting such pathogenic enzymes^8–14^^,^^17–19^ were reported in the last decade^5^^,^^8^^,^^11^^,^^13–15^ and are considered as an alternative to clinically used antibacterials, for which a wide range of resistance was reported in the last decades^5^. Furthermore, pathogenic fungi such as *Candida glabrata*^5^^,^^22^, *C. albicans*^5^, *Cryptococcus neoformans*^5^^,^^22^ or *Malassezia globosa*^23^ also encode for β-CAs which were demonstrated to be druggable, with many interesting inhibitors belonging to various classes reported, some of which also showed some efficacy *in vivo*^5^^,^^22^^,^^23^. Pathogenic protozoans, such as *Plasmodium falciparum* or *Trypanosoma cruzi* also encode for CAs belonging to various classes (η-CA for the first pathogen^7^, and α-CA for the second one^24^) which were shown to be inhibited efficiently by sulphonamide CAIs, with antiprotozoans effects *in vitro* and *ex vivo*^24^. A β-CA was isolated and characterised from the malaria-transmitting mosquito *Anopheles gambiae*, AgaCA^25^, which was proposed as a new drug target for controlling the spread of insect-transmitted diseases. Thus, although the anti-infective field of CA inhibitors (CAIs) was scarcely investigated up until recently, the data presented above and intense research efforts in the last decade demonstrate a great potential for this class of derivatives in the field of anti-infectives targeting bacterial, fungal and protozoan infection as well as the insect pest control.

Recently, our group demonstrated that a widely used antiulcer drug, famotidine (FAM), belonging to the histamine receptor H_2_ antagonists class, also shows significant CA inhibitory properties^26^. Famotidine was in fact assayed as inhibitor of all the catalytically active human (h) CA isoforms, hCA I-XIV and of the two bacterial CAs from H. pylori. Considering the interesting results reported earlier against these enzymes, here we report an inhibition study with famotidine of 20 CAs belonging to the α-η classes from bacteria, cyanobacteria, diatoms, fungi, protozoans and insects.

## 2. Material and methods

### 2.1. Chemistry

Famotidine (FAM) and acetazolamide (AAZ) were commercially available, highest purity reagents from Sigma-Aldrich (Milan, Italy).

### 2.2. CA enzyme inhibition assay

An Sx.18Mv-R Applied Photophysics (Oxford, UK) stopped-flow instrument has been used to assay the catalytic activity of various CA isozymes for CO_2_ hydration reaction^27^. Phenol red (at a concentration of 0.2 mM) was used as indicator, working at the absorbance maximum of 557 nm, with 10 mM Hepes (pH 7.5, for α-, δ-, ζ- and η-CAs) or TRIS (pH 8.3, for β- and γ-CAs) as buffers, 0.1 M Na_2_SO_4_ (for maintaining constant ionic strength), following the CA-catalysed CO_2_ hydration reaction for a period of 10 s at 25 °C. The CO_2_ concentrations ranged from 1.7 to 17 mM for the determination of the kinetic parameters and inhibition constants. For each inhibitor, at least six traces of the initial 5–10% of the reaction have been used to determine the initial velocity. The uncatalysed rates were determined in the same manner and subtracted from the total observed rates. Stock solutions of inhibitors (10 mM) were prepared in distilled–deionised water and dilutions up to 1 nM were done thereafter with the assay buffer. Enzyme and inhibitor solutions were pre-incubated together for 15 min (standard assay at room temperature) prior to assay, in order to allow the formation of the enzyme–inhibitor complex. The inhibition constants were obtained by non-linear least-squares methods, using GraphPad PRISM 3 (GraphPad Software, La Jolla, CA) and the Cheng–Prusoff equation, as reported earlier^26^^,^^28^^,^^29^. All CAs were recombinant proteins produced as reported earlier by our groups^6^^,^^9–14^^,^^17–23^.

## 3. Results and discussion

**Figure 1.**
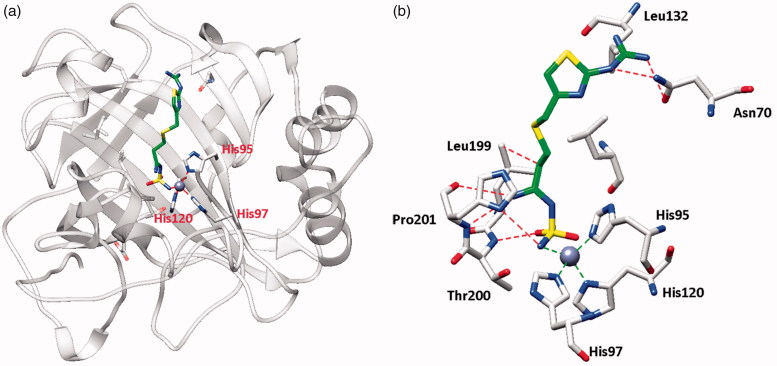
hCA I adduct of famotidine (FAM). (a) Overall structure. (b) Active site details, with the Zn(II) ion (gray sphere), its three His ligands and the inhibitor in green.

**Figure 2.**
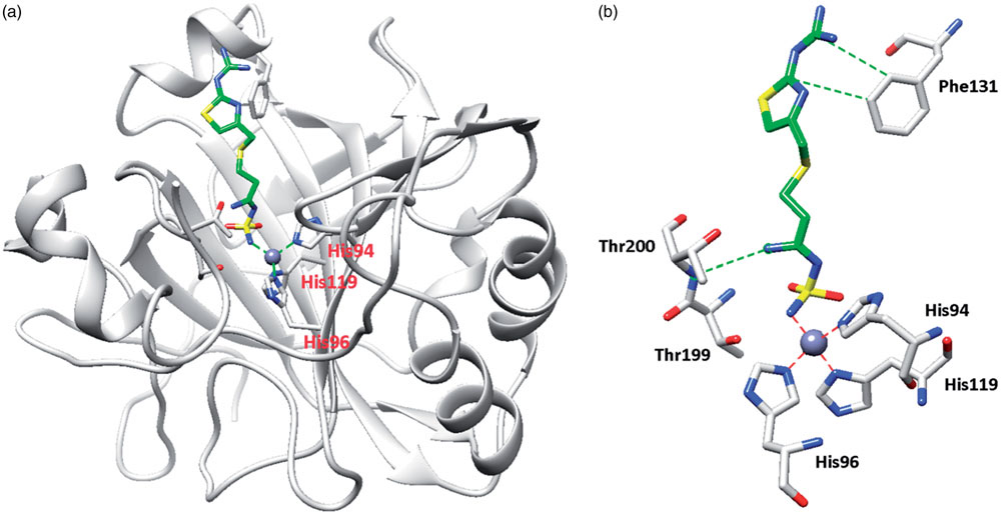
hCA II adduct of famotidine (FAM). (a) Overall structure. (b) Active site details, with the Zn(II) ion (gray sphere), its three His ligands and the inhibitor in green.

The X-ray crystal structures of FAM bound to hCA I (Figure 1) and hCA II (Figure 2) show why this compound effectively inhibits these enzymes^26^. Indeed, FAM has a K_I_ of 922 nM against hCA I and of 58 nM against hCA II^26^.

From the X-ray crystal structures of famotidine bound to hCA I (Figure 1) and hCA II (Figure 2) shown above, it can be seen that the inhibitor makes favorable contacts when bound to both enzymes. The deprotonated sulfamide moiety was coordinated to the zinc ion as the fourth ligand, with the metal in a tetrahedral geometry (the remaining three ligands being residues His95, 97 and 120 in hCA I, and His94, 96 and 119 in hCA II, respectively). The main difference between the binding of the drug to the two isoforms resides in the interaction with the amino acid residue in position 131. Phe131 in hCA II is essential for collocating the inhibitor within the lipophilic half of the active site cavity^26^. On the other hand, the presence of Ser131 in hCA I only led to a loose van der Waals interaction with the famotidine scaffold, which did not force it towards the lipophilic side of the active site, leading thus to two opposite orientations of the scaffold (not shown in Figure 1, see Ref.^26^ for details) and few hydrophobic interactions with the active site. These characteristics reflect the loss of inhibitory potency of famotidine against hCA I compared to hCA II. However, as reported earlier^26^, FAM is an effective inhibitor of the α- and β-CAs from *H. pylori*, with K_I_s of 20.7–49.8 Nm^26^.

Thus, we decided to test the inhibitory effects of FAM (with acetazolamide, AAZ, as standard) against a multitude of CAs of bacterial, diatom, fungal, protozoan and insect origin (Table 1). The following data may be noted from the inhibition constants reported in Table 1:

**Table 1.** Inhibition data against bacterial, diatom, fungal, protozoan, insect and human CAs with famotidine (FAM) and acetazolamide (AAZ) by a stopped flow CO_2_ hydrase assay^22^.


K_I_ (nM)*				
	Organism	Enzyme class	FAM	AAZ
VchCAα	Bacterium	Α	72.3	6.8
VchCAβ	Bacterium	Β	83917	451
VchCAγ	Bacterium	Γ	5321	473
SspCA	Bacterium	Α	230	4.5
SazCA	Bacterium	Α	124	0.9
Rv3274	Bacterium	Β	1265	104
BpsCAβ	Bacterium	Β	8271	745
BpsCAγ	Bacterium	Γ	8293	149
PhaCAγ	Bacterium	Γ	66.8	403
CpsCAγ	Bacterium	Γ	89.0	502
NcoCAγ	Cyanobacterium	Γ	1695	75.8
TweCAδ	Diatom	Δ	408	83
TweCA Cd	Diatom	Ζ	90.7	69
TweCA Zn	Diatom	Ζ	22.1	58
MgaCA	Fungus	Β	42109	40000
Can2	Fungus	Β	107	10.5
CgNce103	Fungus	Β	13.6	11
TcCA	Protozoan	Α	5707	61.6
PfCA	Protozoan	η	142	170
AgaCA	Insect	Β	397	26
hCA I**	Human	Α	922	250
hCA II**	Human	Α	58.0	12.1
				

*Mean from three different assays, by a stopped flow technique (errors were in the range of ±5–10% of the reported values).

**From Ref.^26^.

- The β-CAs from the bacterium *V. cholerae* (VchCAβ) and the fungus *M. globosa* (MgaCA) were quite insensitive to FAM as an inhibitor, with inhibition constants in the high micromolar range (of 42–83 µM). Acetazolamide (AAZ) was also a poor inhibitor of MgCA with a K_I_ of 40 µM.
- Low micromolar inhibitory effects of FAM were observed against the following enzymes: VchCAγ (*V. cholerae*), Rv3273 (*M. tuberculosis*), BpsCAβ/γ (*B. pseudomalei*), NcoCA (N. commune) and TcCA (*T. cruzi*). These enzymes were inhibited with K_I_s ranging between 1265 and 8293 nM, and they belong to the β- and γ-CA classes, except for TcCA which is an α-CA.
- More effective inhibitory effects of FAM were observed for the following enzymes: SspCA and SazCA (from extremophilic bacteria and belonging to the α-CA class), the δ-CA TweCAδ from the diatom *T. weissflogii*, Can2 from *C. neoformans* (fungus), PfCA (from *P. falciparum*) and AgaCA (from the mosquito *A. gambiae*). FAM showed K_I_s in the range of 107–408 nM, and it should be mentioned that these CAs belong to many diverse genetic families (α-, β-, δ- and η-CAs).
- The following enzymes were inhibited by FAM with K_I_s < 100 nM: VchCAα (*V. cholerae*); PhaCA (*P. haloplanktis*); CpsCA (*C. psychrerythraea*); TweCAζ (from the diatom *T. weissflogii*, with Zn(II) and Cd(II) at the active site, respectively); and CgNce103 (from *C. glabrata*). K_I_s in the range of 13.6–90.7 nM were measured for these enzymes belonging to the α-, β-, γ- and ζ-CA classes (Table 1). The enzymes which were the most effectively inhibited by FAM were CgNce103 and the zinc-containing ζ-CA TweCAζ (K_I_s of 13.6–22.1 nM). FAM is in fact a more effective CAI than AAZ against TweCAζ and almost as effective as AAZ against the C. glabrata β-CA.
- The inhibition pòrofiles of FAM and AAZ are rather different against this panel of CAs (Table 1).

## 4. Conclusions

Drug repurposing (or repositioning) started to be considered as an interesting source of new therapeutic/pharmacological agents also in the field of CAIs^30^. Famotidine, an antiulcer drug discovered as a histamine H_2_-receptors antagonist, was recently demonstrated to potently inhibit some human and *H. pylori* CAs belonging to the α- and β-CA genetic families. Here, we prove that famotidine exerts quite interesting CA inhibitory action against bacterial, cyanobacterial, diatom, fungal and protozoan enzymes belonging to the α-, β-, γ-, δ-, ζ- and η-CA classes. For some pathogenic enzymes such as those from *V. cholerae*, *C. albicans* and *P. falciparum* as well as the mosquitoes involved in malaria transmission (*A. gambiae*), the drug showed efficacy in the range of 13.6–397 nM, making it a possible lead or a possible agent for more detailed, *in vivo* investigations.
